# Management of Ebstein Anomaly in the Current Era: The Story of One Fetus and the Collaboration of Many—A Case Report

**DOI:** 10.3390/jcdd11050147

**Published:** 2024-05-09

**Authors:** Ann Kavanaugh-McHugh, Lisa C. Zuckerwise, Stacy A. S. Killen, Emily A. Morris, Rachel T. Sullivan, Mhd Wael Alrifai, David P. Bichell, Melissa Smith-Parrish, Lindsay Freud

**Affiliations:** 1 Thomas. P. Graham Jr. Division of Pediatric Cardiology, Department of Pediatrics, Monroe Carell Jr. Children’s Hospital at Vanderbilt, Vanderbilt University Medical Center, Nashville, TN 37232, USA; stacy.stratemann@vumc.org (S.A.S.K.); rachel.t.sullivan@vumc.org (R.T.S.); 2 Division of Maternal-Fetal Medicine, Department of Obstetrics and Gynecology, University of Virginia School of Medicine, Charlottesville, VA 22908, USA; dcv6dt@uvahealth.org; 3 Division of Neonatology, Department of Pediatrics, Monroe Carell Jr. Children’s Hospital at Vanderbilt, Vanderbilt University Medical Center, Nashville, TN 37232, USA; emily.a.morris@vumc.org (E.A.M.); wael.alrifai@vumc.org (M.W.A.); 4 Department of Cardiac Surgery, Vanderbilt University Medical Center, Nashville, TN 37232, USA; david.bichell@vumc.org; 5 Division of Critical Care Medicine, Department of Pediatrics, Monroe Carell Jr. Children’s Hospital at Vanderbilt, Vanderbilt University Medical Center, Nashville, TN 37232, USA; melissa.smith-parrish@vumc.org; 6 Department of Paediatrics, The Hospital for Sick Children, University of Toronto, Toronto, ON M5G 1X8, Canada; lindsay.freud@sickkids.ca

**Keywords:** Ebstein anomaly, fetal diagnosis, fetal echocardiography, circular shunt, fetal therapies, supraventricular tachycardia, delivery planning, tricuspid valve regurgitation, pulmonary valve regurgitation, case report

## Abstract

Collaborative multicenter research has significantly increased our understanding of fetal Ebstein anomaly, delineating risk factors for adverse outcomes as well as predictors of postnatal management. These data are incorporated into prenatal care and therapeutic strategies and inform family counseling and delivery planning to optimize care. This report details the translation of findings from multicenter studies into multidisciplinary prenatal care for a fetus with Ebstein anomaly, supraventricular tachycardia, and a circular shunt, including transplacental therapy to control arrhythmias and achieve ductal constriction, informed and coordinated delivery room management, and planned univentricular surgical palliation.

## 1. Introduction

Collaborative multicenter research has been essential to expanding our knowledge of the natural history of complex congenital heart disease diagnosed during fetal life. While single-center studies allow us to benefit from the insights and expertise of skilled and thoughtful individuals, they do not have the power of large population studies. In 2015, Freud and colleagues used the insights from previous studies to analyze data from 23 centers to determine risk factors for mortality in fetuses with Ebstein anomaly and tricuspid valve dysplasia [1]. Subsequent analyses of this same data set delineated the potential for pathology progression over the course of pregnancy [2], the significance of associated changes in extracardiac Doppler patterns [3], and perhaps most importantly, the identified predictors for postnatal mortality and circulatory outcomes [4]. The key role of pulmonary regurgitation and circular shunt physiology in the poor outcome for many of these fetuses, outlined in these studies, led to investigation of the use of transplacental indomethacin to constrict the ductus arteriosus and promote antegrade systemic blood flow as a potential adjunct in the care of these patients [5,6,7].

This case report details the course of a fetus whose management reflects the crucial role of these data in guiding our prenatal counseling, medical therapies, and planning for the neonatal period. A central role of the fetal cardiologist is prenatal collaboration with the maternal–fetal medicine team and coordination of skilled multidisciplinary teams for delivery planning, assuring that they are informed and prepared to deliver the resources needed [7,8,9]. This report also details the pathway for the successful transition of an infant with Ebstein anomaly from delivery to postnatal surgical intervention based on prenatal predictors of postnatal physiology.

## 2. Case Description

### 2.1. Prenatal Course

A 34-year-old pregnant patient (Gravida 5, Para 2) was referred to the outpatient Fetal Cardiology Clinic at the Vanderbilt University Medical Center in 2023 for fetal evaluation at 28 6/7 weeks gestation for suspected fetal tricuspid valve abnormality, right heart enlargement, and arrhythmia noted at the time of obstetric evaluation. The patient had elected to proceed with noninvasive prenatal genetic screening through cell-free DNA analysis, which was low risk for trisomies 13, 18, and 21, monosomy X, and triploidy, with predicted male sex. There were no suspected extracardiac abnormalities. The patient was being treated with diet for gestational diabetes mellitus and had no other medical problems. There was no family history of congenital heart disease.

**Figure 1:**
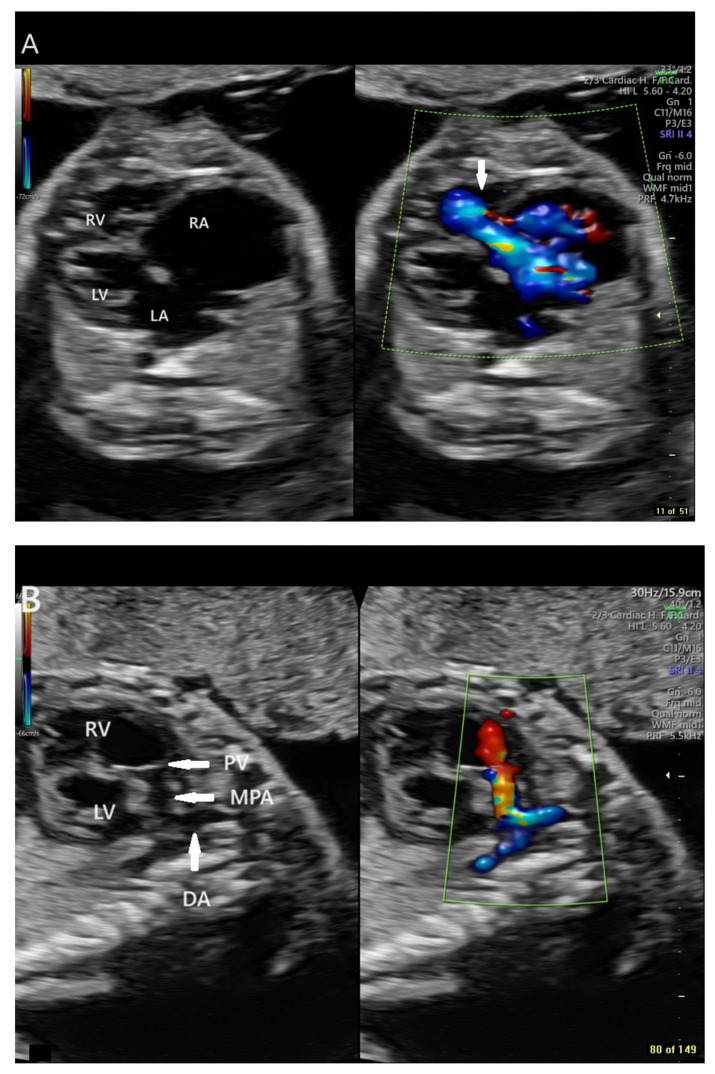
Panel (**A**): Paired fetal echocardiographic apical still frame images showing cardiomegaly, right atrial dilation, tricuspid annular dilation, and right ventricular enlargement at presentation at 28 6/7 weeks gestation. RA = Right Atrium, RV = Right Ventricle, LA = Left Atrium, LV = Left Ventricle. Doppler Color flow mapping superimposed on the panel at right demonstrates severe tricuspid insufficiency (arrow). Panel (**B**): Paired fetal echocardiographic sagittal still frame images of the dilated RV, the normal-sized RVOT, and the reverse-oriented ductus. RV = Right Ventricle, PV = Pulmonary Valve, MPA = Main Pulmonary Artery, LV = Left Ventricle, DA = Ductus Arteriosus. Doppler Color flow mapping in the panel at right demonstrates retrograde flow in the reverse-oriented ductus (blue), retrograde flow in the main pulmonary artery (orange), and marked pulmonary valve insufficiency (red). All images were produced on a GE Voluson E10 Ultrasound System.

Fetal echocardiography was consistent with a diagnosis of Ebstein anomaly with severe tricuspid regurgitation and right atrial (RA) dilation, large tricuspid annulus dimension (Z score +3.5), right ventricular (RV) enlargement (diameter Z score +3.7), mild pulmonary annulus hypoplasia (Z score −2.7), pulmonary insufficiency with retrograde flow in a reverse-oriented ductus arteriosus, and normal branch pulmonary arteries (Figure 1). Tricuspid regurgitation jet velocity was low at 2.3 m/s [1,10,11], consistent with decreased right ventricular performance. At the time of consultation, the fetus was in a virtually incessant supraventricular tachycardia (long RP) with a rate of 203 bpm alternating with several beat runs of sinus vs. atrial rhythm with rates of 150–171 bpm. There was no evidence of fetal hydrops.

The family was counseled regarding the high perinatal mortality for Ebstein anomaly in isolation and the added risk of fetal demise in the setting of fetal tachyarrhythmia. Additional counseling included multiple pathways for postnatal management in the setting of Ebstein anomaly, including both biventricular and univentricular repair. Concern for the need for univentricular repair was emphasized in the setting of a low tricuspid regurgitation velocity, with the possible negative impact of tachycardia on ventricular performance also noted.

**Figure 2:**
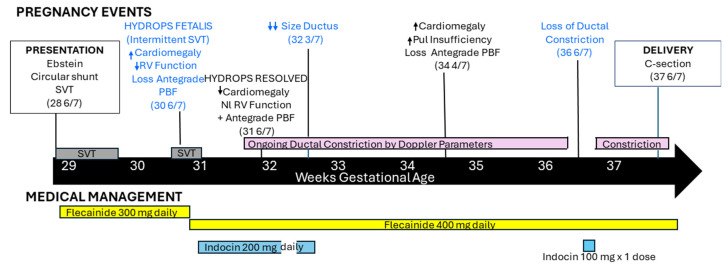
Timeline detailing pregnancy events and medical management during the course of pregnancy. SVT = Supraventricular Tachycardia, RV = Right Ventricular, PBF = Pulmonary Blood Flow, Pul Insufficiency = Pulmonary Valve Insufficiency.

After a discussion of the anticipated overall course for their child and the potential risks and benefits of transplacental therapy, the patient and her partner elected to proceed with antiarrhythmic therapy. The patient was admitted to the obstetric service for the institution of treatment with flecainide 100 mg three times daily with coordinated care by the maternal–fetal medicine and cardiology teams. Baseline evaluation included screening for underlying cardiac disease with electrocardiography and echocardiography, screening for thyroid disease as a potential etiology for fetal tachycardia, and screening for electrolyte abnormalities and vitamin D deficiency as potential contributors to proarrhythmic complications of therapy. Telemetry, electronic fetal monitoring, and serial assessments of ECG and electrolyte status continued until the fetal rhythm was controlled and steady-state drug levels had been achieved. The patient was discharged on flecainide 150 mg twice daily at 29 3/7 weeks gestation with twice weekly cardiology follow-up, as well as twice-daily home Doppler fetal heart rate checks and close concurrent obstetric monitoring (Figure 2).

**Figure 3:**
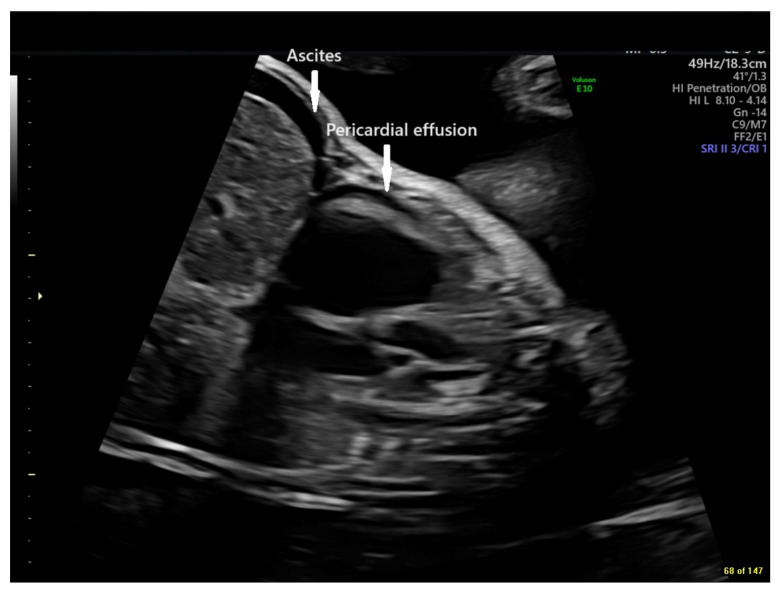
Echocardiographic sagittal still frame image of the fetal chest and abdomen demonstrating ascites and pericardial effusion (arrows) in the setting of early fetal hydrops at 30 6/7 weeks gestation (GE Voluson E10 Imaging System).

At cardiology follow-up at 30 6/7 weeks gestation, fetal echocardiography demonstrated recurrent fetal tachycardia with heart rates of 194 bpm, alternating with sinus vs. atrial rhythm with a rate of 152 bpm. The fetus was in tachycardia approximately 40% of the time. Right ventricular function was decreased when not in tachycardia. There was increasing cardiomegaly (increase in CT area ratio to 0.53 from 0.46), loss of antegrade pulmonary blood flow, continued severe tricuspid insufficiency, and marked pulmonary insufficiency, with retrograde flow in the ductus arteriosus. There was also evidence of hydrops fetalis with fetal ascites and pericardial effusion (Figure 3).

**Figure 4:**
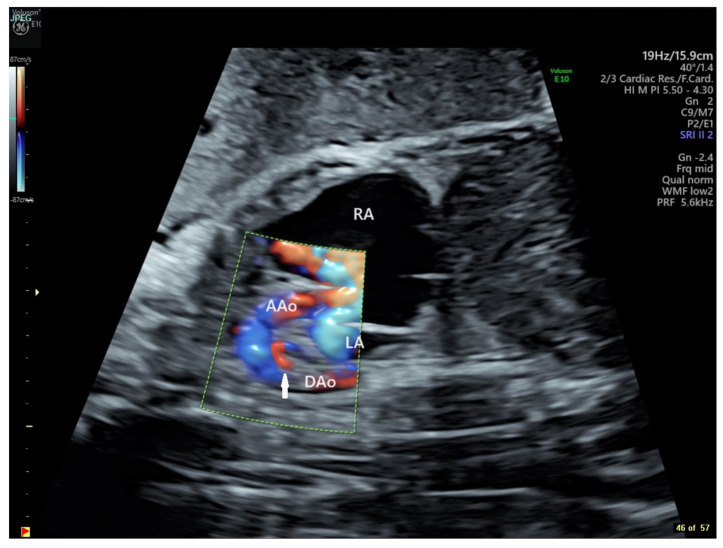
Echocardiographic sagittal still frame image of the fetal thorax with color flow mapping demonstrating retrograde flow in a diminutive ductus arteriosus (arrow). RA = Right Atrium, AAo = Ascending Aorta, LA = Left Atrium, DAo = Descending Aorta (GE Voluson E10 Imaging System).

To address the evolving fetal decompensation in the setting of continued intermittent tachycardia and circular shunt physiology, flecainide was increased to 400 mg divided three times daily. After consultation with the fetal team at The Hospital for Sick Children in Toronto and a discussion of risks and benefits with the family, we agreed to start a trial of indomethacin 100 mg twice daily at 31 1/7 to attempt fetal ductal constriction. On this regimen, the fetal tachycardia was well controlled, and the fetus had evidence of ductal constriction by imaging (diameter Z score −3.4) and Doppler (peak systolic velocity 2.5 m/s; peak diastolic velocity 60 cm/s) by 31 6/7 weeks gestation with associated decrease in CT ratio, restoration of normal right ventricular systolic performance, and resolution of hydrops fetalis. Due to concerns that the ductal diameter had halved in 5 days, indomethacin was held for 48 h and restarted for only 48 h before being discontinued entirely at 32 3/7 weeks, at which time the ductus was difficult to resolve, with best estimates of size in images supplemented by color flow mapping at 2 mm (Z score −6.7) (Figure 4). There was antegrade flow of normal velocity at the pulmonary valve, mild pulmonary valve insufficiency, normal biventricular systolic performance, and no evidence of hydrops fetalis. Tricuspid regurgitation velocity remained low (2.2 m/s), and ensuing discussions with the family regarding postnatal management focused on plans for postnatal univentricular palliation.

Throughout the remainder of the pregnancy, the patient and fetus continued in close follow-up with serial ECG and electrolyte monitoring while on flecainide and with serial fetal echocardiographic evaluations to ensure continued ductal constriction and monitor fetal cardiac compensation. There was loss of evidence of antegrade flow at the pulmonary valve at 34 4/7 weeks gestation and a gradual increase in the pulmonary insufficiency to at least moderate with an increase in fetal CT area ratio to 0.55. When ductal Doppler parameters were no longer in the restrictive range, a single dose of indomethacin was given at 36 6/7 weeks gestation, with restoration of ductal constriction.

**Figure 5:**
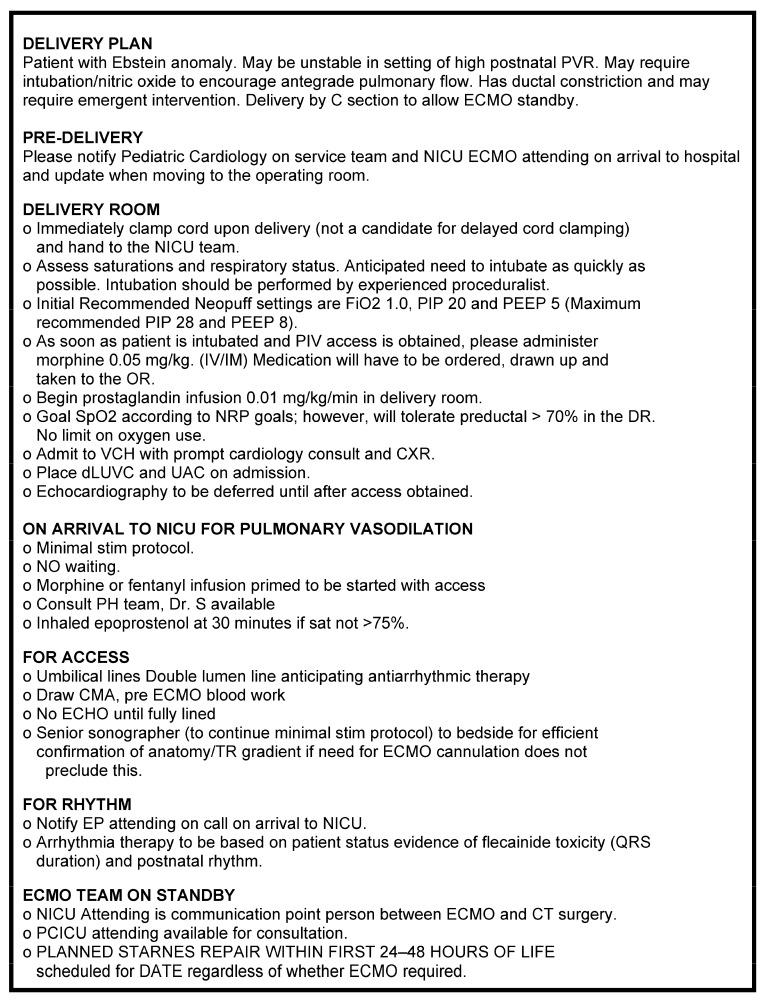
Delivery Plan for multidisciplinary team as saved to Fetal Connections with details of planned management from time of hospital admission of the maternal-fetal dyad until neonatal surgery at 24 to 48 h of age.

During ongoing management of the care of the maternal/fetal dyad, the management team was expanded to include social work and palliative care for family support and representatives from the cardiac intensive care team, neonatology, pulmonary hypertension, and cardiac surgery teams to plan delivery room and postnatal management. The focus of the management plan was the potential for postnatal instability in the setting of high pulmonary vascular resistance and anticipated limited pulmonary blood flow. Plans for expeditious surgical intervention included fenestrated tricuspid valve closure, atrial septectomy, and a Blalock–Taussig–Thomas (BTT) shunt within the first 24 h of life. This detailed management plan (Figure 5) was placed in the patient’s medical record but also set to advance to the fetus’ future chart upon birth through the electronic medical record using EPIC Fetal Connections.

### 2.2. Postnatal Course

Arrangements were made for the Extracorporeal Membrane Oxygenation (ECMO) Team to be on standby, and the patient was delivered by planned cesarean section after spontaneous onset of labor at 37 6/7 weeks gestation. Birthweight was 3160 g, and Apgar scores of 6 and 9 were noted at 1 and 5 min, respectively. The neonate was intubated and started on a prostaglandin infusion and sedative medications in the delivery room, and nitric oxide therapy was started on arrival at the NICU. Postnatal echocardiography confirmed prenatal echocardiographic findings. The neonate proved relatively easy to support with saturations between 90 and 95% until he was taken to the cardiac operating room at 24 h of age for the planned surgical intervention. His subsequent hospital course was complicated by stent placement in the BTT shunt, pericardial effusion and thrombectomy, and feeding issues in the setting of pneumatosis and hematochezia. He underwent G tube placement with Nissen fundoplication after a video fluoroscopic swallow study noted aspiration with oral feeding. The neonate required no antiarrhythmic therapy before or during surgery, though atrial tachycardia requiring treatment with amiodarone emerged on postoperative day 4. Cytogenomic SNP microarray analysis of cord blood showed no copy number changes of known clinical significance and no large regions of homozygosity. As the neonate had no dysmorphic features or extracardiac anomalies, further consultation with our genetics team was deferred. After this difficult initial course, he was discharged to home on POD 104 and is thriving in continued outpatient follow-up in our multidisciplinary Single Ventricle Clinic, which includes nutritional and developmental follow-up and our high acuity home monitoring program. Current plans are for a Bidirectional Glenn shunt as the second stage of his univentricular palliation at four to six months of age.

## 3. Discussion

Our case of successful prenatal diagnosis of severe Ebstein anomaly, in utero medical therapy, and meticulous multidisciplinary care coordination for postnatal course illustrates the importance of delineation of the natural history of congenital heart disease, diagnosed in utero to guide prenatal counseling, targeted therapy, and postnatal planning. The availability of data from other fetuses with this lesion was essential in counseling the family regarding the likelihood of survival, potential in utero therapies, and postnatal surgical options. In addition, the coordinated care of multidisciplinary teams with members both within and outside the institution was essential to the care of this maternal/fetal dyad and the survival of our fetal patient.

The high perinatal mortality of Ebstein anomaly is well recognized, with Freud and colleagues reporting prenatal demise in 19% of fetuses with Ebstein anomaly or tricuspid dysplasia and postnatal death in 32% of liveborn infants for an overall mortality of 45% [1]. This multicenter collaboration examined risk factors for mortality and poor outcomes, which were also shown to be significant in other single-center studies [11,12]. Our patient had two of the four reported risk factors for perinatal mortality at the time of presentation, including diagnosis before 32 weeks gestation and the presence of a circular shunt; other reported risk factors at the time of presentation not shared by this patient include pericardial effusion and marked tricuspid annular enlargement (Z score of 6.8 ± 3). Our fetus shared other risk factors for mortality in the third trimester, including low tricuspid valve regurgitation velocity and lack of antegrade pulmonary blood flow, but did not have decreased left ventricular function or a CT area ratio in a range associated with increased mortality risk.

Presentation with fetal supraventricular tachycardia further increased the risk of fetal demise [8,9,13]. After thoughtful discussion of the relatively low risk to the pregnant patient of transplacental antiarrhythmic therapy when coupled with close monitoring during treatment initiation, most patients consent to medical treatment for their fetus. Collaboration between maternal and fetal medicine and cardiology is essential in transplacental therapy for fetal arrhythmias. Though antiarrhythmic therapy is generally low risk, antiarrhythmic agents can be arrhythmogenic as well. While the risk/benefit ratio generally favors treatment of the fetus, the pregnant person assumes only risk without benefit in agreeing to treatment [8,9,13,14]. Thoughtful discussion of risks and benefits, as well as consideration of overall fetal prognosis, is essential in embarking on therapy. At our institution, patients are evaluated with ECG and echocardiography, with correction of electrolytes and assessment of vitamin D status before treatment [15,16]. Pregnant patients are admitted to our obstetric service for telemetry, electronic fetal monitoring, and monitoring for drug toxicity until they have reached drug steady state on their discharge regimen and fetal rhythm is controlled. Coordinated maternal–fetal medicine and cardiology follow-up include the continued outpatient monitoring for drug toxicity and fetal rhythm abnormalities and subsequent neonatal arrhythmia management [13,14]. Our electrophysiologists consulted in the care of this maternal/fetal dyad prenatally and were then involved in the immediate postnatal assessment of neonatal rhythm and QRS morphology to determine the need for appropriate antiarrhythmic therapy during the period of postnatal decay of fetal flecainide levels.

Of course, in our fetus, the rapid response of the long RP tachycardia to flecainide therapy was not sufficient to prevent progression to the early phases of hydrops, prompting consideration of transplacental therapy with indomethacin for circular shunt physiology. Circular shunt physiology remains an important predictor of perinatal mortality at presentation and throughout pregnancy [1,5,7]. Decisions regarding the institution of transplacental therapy and subsequent monitoring were coordinated by the maternal–fetal medicine and cardiology teams, who remained in communication regarding progress in our weekly multidisciplinary fetal care conferences through direct communication between clinicians and through documentation in the electronic medical record. In the case of our fetal patient, treatment with indomethacin in the early phases of hydrops achieved ductal constriction with resolution of ascites and pericardial effusion, improvement in RV performance, and in fetal compensation as reflected in CT area ratio. Our maternal/fetal dyad was extremely sensitive to indomethacin, necessitating only a short course of this medication. This decreased the likelihood of complications associated with prolonged use of indomethacin during pregnancy, including fetal and postnatal renal failure and oligohydramnios [5,7,17]. Serial echocardiograms to monitor ductal constriction allowed us to recommend a single additional dose later in pregnancy to restore ductal constriction and potentially increase time in utero and the associated increased likelihood of survival with later delivery at a larger size [1].

In the current era, multidisciplinary delivery planning has become central to the prenatal care of families facing a diagnosis of congenital heart disease. Delivery planning includes decisions on timing, mode, and location of delivery [8,9,10,18,19,20], as well as planning for the availability of team members with the skill sets appropriate for the care of the neonate. Multidisciplinary prenatal discussions of this patient prenatally resulted in a thoughtful delivery plan. Progress in communication through the electronic health record (EHR) has allowed wide and efficient dispersal of delivery plans. At our institution, we developed “Fetal Connections”—a novel EHR-integrated application that automatically transmits the prenatally agreed-on plans for neonates from maternal to newborn charts [21]. This application secures plan visibility by all clinical stakeholders and ensures timely actions by neonatal providers. Recently, we added a layer of active decision support that triggers when an eligible pregnant mother is admitted to the hospital. It leads to the notification of multidisciplinary team members to prepare for the special resources needed during and after the delivery of a potentially critically ill infant.

In drafting our delivery plan, we focused on the anticipated postnatal physiology of the neonate, the availability of all necessary services, and rapid transition to the operating room for further stabilization. The low tricuspid regurgitation velocity in fetal life, confirmed postnatally, informed our planning of postnatal medical and surgical management. Our team was aware that the physiology that stabilized the fetus in utero would be difficult to support postnatally. Our patient with Ebstein anomaly would be considered LOC 4 (level of care 4) [19]. Delivery was by cesarean section, after counseling and shared decision-making with the family to optimally coordinate with the ECMO team and ensure that all team members were ready to support the infant immediately at birth.

In the setting of a right ventricle with little apparent ability to generate antegrade pulmonary blood flow and a restrictive ductus potentially limiting retrograde perfusion of the lungs, the team was sufficiently concerned regarding the potential inadequacy of pulmonary blood flow that our NICU “pulmonary hypertension” protocol was planned and initiated with the use of intubation, sedation, and low stimulation infant handling [22,23,24]. Inhaled nitric oxide therapy was started on arrival at the ICU [22,25], and though our pulmonary hypertension team was on standby, as was the ECMO team, adequate pulmonary blood flow proved relatively easy to achieve. Importantly, tricuspid regurgitation velocity as a surrogate of right ventricular systolic performance is a critical fetal and neonatal predictor of postnatal success with biventricular repair. The low tricuspid valve velocity did not support plans for biventricular repair, instead favoring univentricular palliation with an RV exclusion procedure and BTT shunt, decreasing RV volume and unfavorable ventricular interactions and improving LV filling and performance. The parents did not meet their child’s surgeon prenatally, as the infant was delivered the day before the planned consultation. However, prenatal discussions with the surgical team reviewing the fetal predictors of postnatal physiology allowed us to agree on the surgical plan for postnatal atrial septectomy, fenestrated closure of the tricuspid valve, and creation of a BTT shunt, as well as to be able to proceed with the expeditious institution of surgical palliation postnatally.

Further advances in the care of patients diagnosed prenatally with rare lesions will require continued multicenter and multidisciplinary collaboration following the fetal and postnatal courses of these children. The Fetal Ebstein anomaly and tricuspid valve dysplasia (FEAT) Registry is one such multicenter collaboration that aims to follow prenatally diagnosed infants with Ebstein anomaly and tricuspid valve dysplasia lifelong, examining outcomes including response to interventions, overall survival, and neurodevelopmental outcomes. The collaboration of many to expand our understanding and inform our management remains essential to our ability to move each individual patient and our entire field forward.

## 4. Patient Perspective

Added with patient’s permission according to Care Guidelines: “To begin, there are two points that encompass so much of what has brought our family through this season with our son. First, our faith in Jesus Christ and knowing our little baby boy is in His hands has kept us grounded in knowing our God is in total control. Secondly, being placed in the care of the most wonderful medical professionals who value human life to the extent they have rallied around us to fight for our baby boy’s life, even when things seemed so bleak. Receiving the reports where we were on the verge of losing our baby boy is the most difficult news we have ever received, but with this news there was also presented a very detailed plan of care that carried with it the potential of saving his life. Although at one time the plan of care was largely experimental, knowing there was a possibility it could save our baby boy’s life, moving forward with the plan of care presented by our doctors was an easy decision when the alternative was certain loss of life. Taking medicines that I didn’t need, but were vital for our unborn son’s survival was something I had never experienced before. Although it was a little unnerving not knowing how my body would respond, my only thought was helping our son have his best chance of survival. As a patient and as a family unit, we could not have felt more cared for by the amazing medical staff that surrounded and supported us. Our son is now six months old and thriving. If there is any advice we could give to families who are going through something like this it would be to never give up and take a chance on a ground-breaking treatment plan, even if it seems impossible, to give your baby their best chance”.

## 5. Conclusions

This case report emphasizes the importance of understanding fetal natural history to advance our understanding of lesions and support our counseling of families regarding treatment options and likely courses for their pregnancies and children. Multicenter natural history studies are critical in amassing data on rare lesions. Data from these multicenter studies are critical to expanding our understanding of perinatal physiology and for appropriate planning of the needs of infants and families at the time of delivery and beyond. Communication between teams both within and outside our institutions is essential to optimizing care and can be supported by novel EHR functionalities. Long-term multicenter studies will be essential for further improving our understanding of these lesions and improving outcomes.

## Data Availability

Data are recorded only in this publication.
